# Quadra Sense: A Fusion of Deep Learning Classifiers for Mitosis Detection in Breast Cancer Histopathology

**DOI:** 10.3390/diagnostics16030393

**Published:** 2026-01-26

**Authors:** Afnan M. Alhassan, Nouf I. Altmami

**Affiliations:** Department of Computer Science, College of Computing and Information Technology, Shaqra University, Shaqra 11961, Saudi Arabia

**Keywords:** normalization, histogram equalization, deep learning (DL), normalization, CNN, Mito-Quartet

## Abstract

**Background/Objectives**: The difficulties caused by breast cancer have been addressed in a number of ways. Since it is said to be the second most common cause of death from cancer among women, early intervention is crucial. Early detection is difficult because of the existing detection tools’ shortcomings in objectivity and accuracy. Quadra Sense, a fusion of deep learning (DL) classifiers for mitosis detection in breast cancer histopathology, is proposed to address the shortcomings of current approaches. It demonstrates a greater capacity to produce more accurate results. **Methods**: Initially, the raw dataset is preprocessed by using a normalization by means of color channel normalization (zero-mean normalization) and stain normalization (Macenko Stain Normalization), and the artifact can be removed via median filtering and contrast enhancement using histogram equalization; ROI identification is performed using modified Fully Convolutional Networks (FCNs) followed by the feature extraction (FE) with Modified InceptionV4 (M-IV4), by which the deep features are retrieved and the feature are selected by means of a Self-Improved Seagull Optimization Algorithm (SA-SOA), and finally, classification is performed using Mito-Quartet. **Results**: Ultimately, using a performance evaluation, the suggested approach achieved a higher accuracy of 99.2% in comparison with the current methods. **Conclusions**: From the outcomes, the recommended technique performs well.

## 1. Introduction

Breast cancer (BC) is the second most common cause of cancer-related death among women, according to the World Health Organization (WHO). The importance of early diagnosis cannot be overstated. About 500,000 breast cancer patients have died, and many more are diagnosed each year. These numbers should climb sharply. Ineffective treatment and late diagnosis reduce patients’ life expectancy and quality of life. Females aged 20–59 globally die from this cancer. Early detection increases breast cancer survival to 80% [1,2]. The WHO notes that it affects women after puberty in any country at any age, with rates rising with age. The cancer is still hard to detect, so a newly diagnosed patient must be evaluated using systemic grading. There are five phases overall. Stages I through IV are characterized by the size of the tumors, which can be invasive or noninvasive, and could have spread; however, survival rates decrease. Breast cancer mortality decreases, and survival improves with early diagnosis and detection. It is a major health risk for women [3].

Several methods are used to identify breast cancer (BC), including breast temperature monitoring, three-dimensional ultrasound, CT, Positron Emission Tomography (PET), MRI, and X-ray mammography. However, the diagnosis of pathology is often considered the definitive and most reliable criterion. In order to enhance observation and analysis, it is customary to subject the excised tissues to staining, with H&E staining being a prevailing approach employed for this purpose. Eosin is used to provide a pink color to the cytoplasm, whereas hematoxylin is employed to produce a dark purple hue in the stroma and other structures [4]. Breast cancer patients worldwide have been assessed using the Nottingham Histopathology Grading (NHG) system, widely regarded as the most dependable prognostic predictor. In addition to the processes of tubule formation and nucleus pleomorphism, the mitotic count holds significant prognostic value within the NHG system. Histopathologists employ a light microscope as a standard procedure to enumerate cells undergoing mitosis [5].

Breast cancer mitotic detection may be critical to cancer development. Understanding the mitotic count’s importance can help assess tumor aggressiveness and proliferation [6]. Mitosis has five stages: interphase, prophase, metaphase, anaphase, and telophase. It is hard to spot in images because it appears as tiny objects with several shapes. Different mitosis stages modify the nucleus’s form. Although two separate nuclei are formed during the telophase of mitosis, these are not yet fully developed cells. It takes a long time to diagnose mitosis in every cell.

Advanced image processing and pattern recognition algorithms can automate this process, saving time, money, and labor [7]. Understanding biological processes in medicine requires microscopic images of human organs and tissues. For microscopic image analysis, classifying images such as tissues, organs, etc., is essential. Applications classify images from microscopy [8]. For the diagnosis of cancer, mitotic cells in histological images represent a proliferative sign.

Thus, accurate mitotic cell detection is crucial for the detection of cancer [9]. Histological examinations are performed in pathology labs to study tissue disease expression. Unlike histopathological image analysis, fine needle aspiration cytology investigates cell structure and characteristics [10]. Automatic mitotic detection in histological image analysis is difficult. Each phase has a unique mitotic figure shape. The possibility of misdiagnosis increases with H&E-stained images, as they can show mitotic figures and other similar features such as dense nuclei and lymphocytes. Therefore, pathologists may miss important mitotic figures due to slide preparation changes (staining method, concentration, percentage, etc.). Manual identification is monotonous and susceptible to scrutiny variance [11].

Due to human workflow disadvantages, mitotic nuclei counting must be automated to improve pathologists’ judgment. Computer-aided pathology is a result of the digitisation of histopathology and recent developments in medical (IP) image processing and machine learning (ML). This field has created automated methods for categorizing tumorous tissues, identifying biomarkers, and recognizing nuclei [12]. CAD systems are one of the fastest-growing fields of DL-based medical diagnostics. At present, professionals use such technologies to discover and classify problems. Selection of meaningful features is critical in classification model learning because CAD system performance depends on feature quality and classifier learning capacity [13]. Advanced artificial intelligence technology allows digital images to produce more accurate and reliable results. Machine learning-based feature extraction, selection, and classification are used to analyze digital histopathology pictures. The feature extraction stage extracts unique features from input pictures. Deep learning is effective at automatically learning from extracted features and performing regression and classification. It has numerous hidden layers, a new training paradigm, and is based on the traditional Neural Network (NN): pattern recognition, ML, and computer vision. These traits boost classification [14,15]. The main contributions of this work are summarized as follows:

▪ A hybrid mitosis detection framework that integrates deep learning-based feature extraction with handcrafted texture, color, and shape features, improving robustness and discriminative capability.
▪ A modified FCN-based ROI identification module to accurately localize mitotic regions and reduce background interference.
▪ A Modified Inception V4 network for deep feature extraction, combined with a Self-Improved Seagull Optimization Algorithm (SA-SOA) for optimal feature selection.
▪ A hybrid Mito-Quartet classification model that fuses CNN, optimizes RNN, and utilizes an attention mechanism and Bi-LSTM to enhance detection accuracy.
▪ Comprehensive experimental evaluation and comparison with existing learning-based methods using standard performance metrics.

The remainder of this paper is organized as follows. Section 2 reviews related work in mitosis detection and histopathological image analysis. Section 3 describes the proposed hybrid methodology in detail. Experimental results and discussion are presented in Section 4, followed by conclusions in Section 5.

## 2. Related Works

To predict breast cancer, a hybrid CNN technique is suggested by Nalini Sampath et al. [16]. To increase accuracy, this hybrid CNN approach combines transfer learning (TL) with the Sine–Cosine Algorithm (SCA). The VGG 16 architecture uses sine–cosine technique hyperparameters, and transfer learning is used for the final convolutional layers. The accuracy in the proposed approach is increased, achieving 96.9% accuracy, demonstrating the model’s efficacy.

Muhammad Sadiq Amin et al. [17] proposed a FabNet model using an accretive network architecture for classification accuracy in histopathology images. The model uses deep integration to mix elements across layers, achieving greater accuracy and fewer parameters. The model outperforms alternatives in detecting malignant tumors.

The Hummingbird algorithm with TL-based mitotic nuclei classification (AHBATL-MNC) was used by Areej et al. [18] to create a technique for histopathologic breast cancer images. They used the PSP Net model, XG Boost model, and Res-Net model, with the AHBA technique to improve classification performance. Simulation results showed enhanced outcomes.

To accurately identify breast cancer, Paterl et al. [19] used a deep network with a graph-based adaptive regularized learning network (GARL-Net). The network, DenseNet121, achieves a 99.5% accuracy rate by using a complement cross entropy loss to address misclassification and enhance learning efficiency after being trained by TL.

Deshmukh Pramod Bhausaheb et al. [20] suggested an approach using histopathological images by a Deer–Canid-based deep CNN. The V-net architecture was used to segment images while maintaining originality. Deer–Canid optimization reduced classification time and achieved optimal global solutions. The proposed deep CNN achieved high performance, demonstrating its superiority in the field.

To diagnose breast cancer datasets and distinguish between benign and malignant forms, Mohammed Al-Jabbar et al. [21] created three algorithms. The first approach makes use of an ANN that combines features from ResNet-18 and VGG-19. The proposed method combines features extracted from VGG-19 and ResNet-18, both before and after applying PCA. An ANN with hybrid attributes, such as VGG-19 and handcrafted features are used in the final method. The accuracy of this hybrid feature is 99.7% for the binary classes data set and 97.3% for the multi-class data set.

Ramkumar et al. [22] presented a framework to highlight the importance of early detection in treating BC. They employed machine learning to create fully automated breast cancer detection tools, using Support Vector Classifiers (SVCs) and KNN classifiers. The study found that SVC outperforms KNN in diagnosis with a lower error rate (94.12%), highlighting the need for early detection and effective treatment methods. This approach can help prevent the spread of chronic conditions and increase global death rates.

Sajiv et al. [23] recommended an approach for detecting BC. Researchers use classification to analyze large medical data volumes and discern between benign and malignant tumors without invasive surgery. The Kaggle database’s cancer data is utilized for training this method with an accuracy of 85%. When combined with other detection methods like MLP, enhances the chance of early cancer diagnosis. The suggested algorithm focuses on disease detection and diagnosis.

Gül [24] suggested two CNN-based and local binary pattern (LBP)-based techniques for high-performance preliminary diagnosis of breast cancer histopathology images. The 20-layer CNN model is named quad star LBP (QS-LBP) since it is based on the suggested LBP and has a star-like topology. The most popular machine learning algorithms, Random Forest and Optimized Forest, were employed to examine the histopathology images that had been enhanced by the QS-LBP approach.

Singh et al. [25] presented a unique framework that integrates depth-enhanced semantic information with multi-scale RGB characteristics to detect tumors in histopathology pictures. The Adaptive Multi-Scale Fusion Module (AMSF), Semantic Depth Integration and Calibration Module (SDICM), and Depth-Guided Tumor Detection Module (DG-TDM) are the three main components of the approach. The AMSF creates a fused feature map that captures occlusion information and border relevance by combining RGB histopathology image channels, binary masks, and multi-scale convolution outputs employing attention processes. By combining RGB, semantic, and sparse-depth data through bidirectional feature aggregation, SDICM creates dense depth maps that improve edge clarity and spatial continuity.

Haq et al. [26] proposed advanced DL architectures like CNN, generative adversarial networks (GANs), autoencoders, deep belief networks (DBNs), extreme learning machines (ELMs), transformer-based models like Vision Transformers (ViTs), transfer learning, attention-based explainable AI techniques, and multimodal integration, which were evaluated to address these diagnostic challenges. Identify the primary developments, limitations, and opportunities for additional research. We also look at the critical roles that XAI, multimodal integration, and synthetic data augmentation play in enhancing diagnostic accuracy, model interpretability, and clinician trust, all of which ultimately lead to more effective and customized patient care.

The medical image community lacks sophisticated manual annotations due to their high cost and time-consuming nature. AI offers objective analysis capabilities in histopathology detection, preventing subjectivity. Deep learning techniques can improve pathologists’ decisions and productivity. AI has achieved significant success in medical image analysis, delivering strong classification performance due to extensive labeled data.

## 3. Materials and Methods

**Figure 1:**
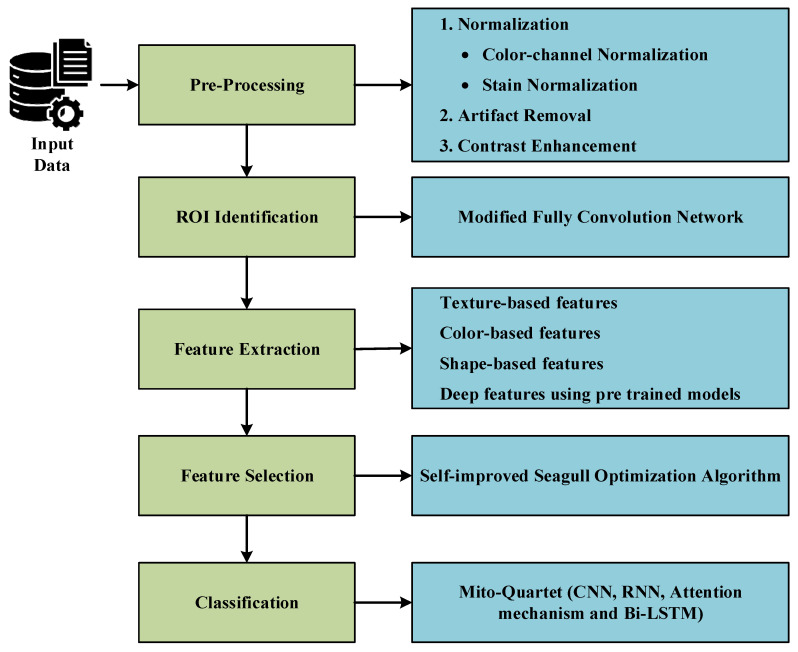
Block diagram of the proposed model.

Conventional breast cancer screening and diagnostic systems have known challenges in terms of precision, objectivity, and scalability, especially as far as the detection of mitosis in breast cancer histopathological images is concerned. To meet all these challenges, the research work proposes the “Quadra Sense” system, which is a hybrid deep learning and feature-based model for the detection of mitotic figures in breast cancer histopathology images. Contrary to the conventional deep learning models, the proposed model combines the high-level feature extraction capability of deep neural networks with feature extraction and optimization processes for the exploitation of semantic as well as domain-specific knowledge. The raw breast cancer histopathological images are preprocessed initially using color channel normalization, stain normalization, median filtering, and histogram equalization to improve the quality of the images and remove noise from them. A modified Fully Convolutional Network (FCN) is consequently used for the detection of the region of interest (ROI), such that further processing is performed for the identification of the mitotic figures within the ROI areas. From the localized ROIs, the high-level features are extracted through the application of the Modified Inception V4 (M-IV4) network. Alongside this, the handcrafted features, including the morphological features based on the texture, color, and shape, are extracted. The blended feature set is optimized utilizing the Self-Improved Seagull Optimization Algorithm (SA-SOA) for the selection of the most dominant feature subset. As a final step for the mitosis classification, the Mito-Quartet combines the CNN, the optimized RNN, the attention mechanism, and the Bi-LSTM for the identification of the feature set dependencies based on spatial and temporal features. Figure 1 depicts the composite process implemented by the hybrid approach.

### 3.1. Pre-Processing

An essential phase in transforming the unprocessed dataset into the ideal format. Better segmentation results from an efficient preprocessing step led to an increase in classification accuracy. The dataset can contain some noisy signals and redundant data. Considering the dataset as DS0, DS1, DS2, …, DSn. The recommended method uses a few pre-processing techniques, which will be thoroughly discussed as follows.

#### 3.1.1. Normalization

This process entails manipulating the original data through rescaling or transformation methods to ensure that each characteristic makes a uniform contribution. There are various types of normalization that are in existence. Two normalization procedures are utilized in this research.

##### Color Channel Normalization (Zero-Mean Normalization)

The values of a feature are scaled using this method by guarantying standard deviation of one and an average value of zero. It is accomplished by subtracting the feature mean from each value, then dividing each individual value by the standard deviation.

(1) $$M_{norm}\left(DS\right)=\frac{M-\mu }{\sigma }=D$$

where $M_{norm}$ → normalized data, M → input data σ → standard deviation, μ → mean:

(2) $$\mu =\frac{1}{n}\sum_{i=1}^{n}M_{i}$$

(3) $$\sigma =\sqrt{\frac{1}{n-1}\sum_{i=1}^{n}{\left(M-\mu \right)}^{2}}$$

##### Stain Normalization (Macenko Stain Normalization)

The staining approach often employed in medical diagnostics involves the utilization of hematoxylin, which selectively imparts a blue-purple hue to nucleic acids, and eosin, which imparts a vivid pink coloration to proteins. Additional variations occur due to staining chemicals that exhibit somewhat different colors as a result of their differing degrees of light absorption. In accordance with established conventions, it is customary to convert all color values into their respective optical density (OD) values.

(4) $$OD\;(D)=-{log}_{10}\left(I\right)$$

The RGB color vector is denoted as I, with each component normalized to the range of [0, 1]. After identifying the appropriate vectors, a straightforward color deconvolution method is employed to convert the visual representations into numerical proportions.

(5) OD=VS

(6) $$S=V^{-1}OD$$

Here, S represents the saturations of each stain and V represents the matrices of the stain vectors.

The class of unsupervised normalization techniques includes Macenko Stain Normalization. To estimate the stain vectors of H&E in the whole slide image (WSI), the program first applies the Singular Value Decomposition (SVD) technique, paying particular attention to the input image’s non-background pixels. To adjust differences in intensity caused by the stain’s original strength, staining technique, etc., the algorithm then applies a correction.

In order to ensure that all normalized images have comparable color properties following stain normalization, the image is finally reflected onto a specified image. The foundation of the technique is the idea that each pixel’s color (represented by RGB channels) is an integration of 2 H&E stain vectors (sv) that must be calculated. Algorithm 1 explains the SVD–geodesic method for obtaining stain vectors. Through this processing, we can release values that are not as crucial to maintaining the image’s quality while still preserving the singular values that the image needs.
**Algorithm 1.** SVD approach for obtaining sv.Input: RGB Slide, Output: Optimal Stain VectorsConvert RGB to OD using a known transformation Eliminate data points with OD intensity below β. Perform SVD on the OD tuples to reduce dimensionality. Create a plane using the two greatest values’ SVD directions. Project OD tuples onto the plane and normalize to unit length. Compute the angle of every point on the planeIdentify robust extremes by determining the $\alpha^{th}$ and (${100-\alpha )}^{th}$ percentiles of the angle. Use α = 1 for robust results. Transform high values back to OD space

#### 3.1.2. Artifact Removal

Signal and image processing sometimes need smoothing chaotic signals while preserving edge information. Median filtering is a popular smoothing approach. The median of an odd-numbered group is the middle element after sorting. In normal median filtering, an odd-sized window is shifted along the sampled signal or picture values. The median of the window’s elements is calculated for each position and written at the output pixel equal to the window’s central element. As the window dimension is constant, the count of entering and outgoing objects is equal. Filter mask dimensions must be odd. Mask sizes are 3 × 3, 5 × 5, or 7 × 7. Often, a minimal one is best. They eliminate impulsive noises remarkably well, smooth out transient fluctuations in signal strength (such as noise), preserve edge information in the filtered signal, and can be executed by uncomplicated digital asymmetric processes.

#### 3.1.3. Contrast Enhancement

Dynamic range, or the range of brightest to darkest pixel intensities, determines the contrast of an image. There are various applications for using contrast enhancement techniques to improve low-contrast images. Histogram equalization (HE) is a commonly utilized technique. Gray levels are mapped using the input gray level probability distribution. Contrast is enhanced by stretching and flattening the image’s histogram. Histogram equalization (HE) is used in radar IP and medical IP. The probability density function $P(I_{x})$ for image I is stated as

(7) $$P\left(I_{x}\right)=\frac{n_{x}}{n}$$

The level $I_{x}$ input is used $n_{x}$ times, and here, x=0,1,…,L−1. The total number of samples is denoted by n.

The Cumulative Density Function (CDF) is expressed as

(8) $$c\left(i\right)=\sum_{j=0}^{x}P\left(I_{j}\right)$$

Here, $c\left(I_{L-1}\right)=1$ (constant) and $I_{x}=i$ for x=0,1,…,L−1. According to CDF, the transform function $f\left(i\right)$ is expressed as

(9) $$f\left(i\right)=I_{0}+\left(I_{L-1}-I_{0}\right)c\left(i\right)$$

$S=\left\{S\left(q,r\right)\right\}$, the output of histogram equalization (HE), is given below

(10) $$S=f\left(I\right)=\left\{f\left(I(k,j\right)|\forall I(k,j)\in I\right\}$$

The dynamic range expansion results in great histogram equalization (HE) performance by boosting an image’s contrast.

### 3.2. Region of Interest (ROI) Identification

**Figure 2:**
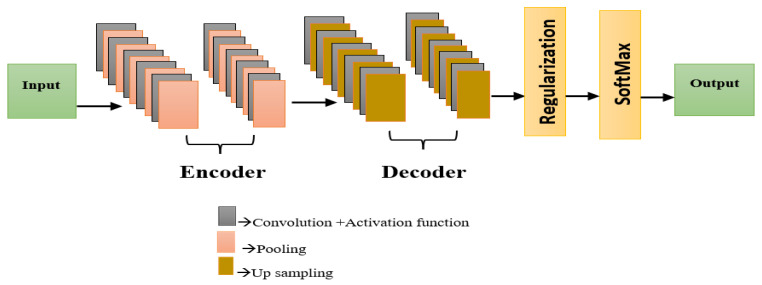
Regularized FCN.

In medical image analysis, the localization of the region of interest (ROI) is of extreme importance to successfully identify or diagnose various types of diseases, specifically for mitosis detection, which has a few target objects, as well as a small size. In this research, localization of the region of interest is carried out by using a regularized Fully Convolutional Network (FCN) based on the encoder–decoder framework, as shown in Figure 2. The ground truth of ROI is obtained from mitosis annotation provided by experts in the MITOSIS 2012 benchmark, which represents all marked points of mitosis by generating a binary ROI mask centered at the centroid of mitotic points, using a fixed-size region. Pixels representing the mitotic areas are marked as the foreground pixels, whereas the other areas of the tissue are marked as the background pixels to supervise the fine details of the pixels during the training phase. The FCN model learns the discriminative representation of the pixels from the histopathological image during the supervised learning phase with the histopathological image patches and their respective ROI masks. As the decoding process requires the reproduction of the spatial details within the mitotic areas to accurately identify the promising mitotic areas, the ROI probability maps generated during the decoding phase are passed to the next stage of processing.

#### 3.2.1. Convolution Layer (CL)

These layers extract and map features from the input, which is defined as

(11) $$conv=\left(filt,\;ReLU\right)$$

#### 3.2.2. Activation Layer

The ReLU activation function used for image transformation is nonlinear. This converts feature maps into the system for effective training and learning. ReLU is given as

(12) $$max\left(0,x\right)=\left\{\begin{array}{c} x,\;x\geq 0\\ 0,\;x<0 \end{array}\right\}$$

#### 3.2.3. Pooling Layer

The pooling layer diminishes the dimensions and resolution of the derived feature maps. It mitigates complexity and the inclination towards overfitting, as well as minimizes the computational processing time. The layer utilizes the equation provided below in (13):

(13) $$Layer=Max\;Pooling\;\left(Pool\;size\right)$$

Second, the decoder learns data spatial features for recovery and border location. The input function map encoder stage size is restored. Convolution layers, ReLU activation, and up-sampling layers are in each decoder block. It recovers spatial features and boundary positions while the convolution layer extracts features. Encoder and decoder sections have a short skip link. The skip connection merges and concatenates the encoder and decoder convolution layer outputs. This enhances feature map restoration. Final decoder output goes to the next model stage.

#### 3.2.4. Regularization

The decoder’s final output undergoes regularization. It indicates approaches for calibrating ML models to reduce the loss function (for example Dice loss function) and avoid overfitting or underfitting. Batch normalization is applied in our situation. It is a normalization method used within a Neural Network’s layers as opposed to in the raw data. It facilitates accelerating training. To fit all pixels into a tolerable range, normalization can be utilized. Weights (the components of its filters) may become too large, producing data with pixels that are dispersed over a wide range. Batch normalization efficiently generates a new mean and standard deviation for every pixel in every feature map in a convolution layer. This technique is completed by normalizing the z-score, multiplying the results by an arbitrary scale parameter (alpha), and then adding another arbitrary offset value (beta).

The following is the specification for the batch normalization.

(14) $$m^{\prime }=\left(\frac{\left(m-\mu \right)}{\sigma }\;*\alpha \right)+\beta$$

The regularized entropy is given as

(15) $$m^{\prime }=\left(\frac{\left(m-\mu \right)}{\sigma }\;*\alpha \right)+\beta *H$$

where $m^{\prime }$ → batch normalized value, m → feature map (fm) element, μ → mean, H → entropy, and α, β → learnable arbitrary parameters.

Entropy H is used to quantitatively gauge the degree of presence or absence of a set to define an ambiguity.

(16) $$H=plogp^{-1}+\left(1-p\right)\log{\left(1-p\right)}^{-1}$$

The probability p is given as

(17) $$p=\frac{1}{s}\sum_{i=1}^{s}f\left(m_{i}\right)$$

where s → number of elements.

#### 3.2.5. Fully Connected (FC) Layers

The SoftMax classifier predicts pixel classes from the regularized output, as shown in Equation (18).

(18) $$Softmax\left(y\right)=\frac{e^{y}}{\sum_{j}e^{yj}}$$

#### 3.2.6. Output Layer

The network concludes at this layer, yielding the final output.

### 3.3. Feature Extraction

The next stage of the model is the FE procedure. FE uses the information from the previous step to carry out the operation. FE approaches are utilized to extract features that will be useful for image classification and identification. Features are essential in the field of image processing. The procedure of converting a collection of attributes from pre-processed data is known as FE. It is performed using the following features, which are explained in detail.

#### 3.3.1. Texture-Based Features

Texture is a crucial characteristic for various sorts of images seen in nature, including medical images and sensor images.

##### Local Binary Pattern (LBP)

While texture features employ groups of pixels, color features only use individual pixels. Each pixel in the feature maps has a Local Binary Pattern (LBP) calculated for it. It encodes the results in binary by comparing the data.

It takes the segmented objects’ surface features, such as patterns, edges, or edges, and extracts texture information from them. The LBP is a useful nonparametric operator for specifying localized picture properties.

An ordered binary set known as LBP is created by comparing the gray values of the central pixel’s $\left(x_{c},y_{c}\right)$ to those of its eight neighbors. Consequently, the LBP code is expressed in decimal form as an octet value,

(19) $$z=LBP\left(x_{c},y_{c}\right)=M\left(\sum_{n=0}^{7}S\left(i_{n}-i_{c}\right)2^{n}\right)$$

Here, the gray value of the center pixel $\left(x_{c},y_{c}\right)$ is denoted by $i_{c}$. The gray value of each pixel’s eight neighbors is denoted by $i_{n}$. Following the transformation, the outcome is presented as

(20) $$S\left(i_{n}-i_{c}\right)=\left\{\begin{array}{c} 1\;;\left(i_{n}-i_{c}\right)\geq 0\\ 0\;;\left(i_{n}-i_{c}\right)<0 \end{array}\right.$$

##### Gabor Filter

The use of Gabor is a different technique for obtaining texture data. Extracting texture features from images, Gabor filters have been widely employed. By defining the center frequency (freq.) and orientation parameters, the Gabor filter is specifically made to sample the whole freq. domain of a picture. Each wavelet collects energy in a particular direction and freq, resulting in a localized freq. that serves as a feature vector. Thus, from this collection of energy distributions, texture features can be derived.

The formula for a two-dimensional Gabor function $g\left(x,y\right)$ is denoted as

(21) $$g\left(x,y\right)=\frac{1}{2\pi \sigma_{x}\sigma_{y}}e\left[-\frac{1}{2}\left(\frac{x^{2}}{\sigma_{x}^{2}}+\frac{y^{2}}{\sigma_{y}^{2}}\right)+2\pi \theta W\right]$$

where $\sigma_{x}$, $\sigma_{y}$ are the scaling parameters of the filter, W is the center frequency, θ is the orientation.

#### 3.3.2. Color-Based Features

Hue, saturation, and intensity are used by HSI to describe the characteristics of color. In this color space, the chromatic information is represented by the letters H and S, whereas the luminance information is represented by the letter I, which has nothing to do with color.

The color itself is represented as an angle between [0, 360] by the Hue component in the HSI color space. With a range of [0, 1], the saturation component shows the extent to which the color is tainted with white. With 0 representing black and 1 representing white, the intensity component has a range of [0, 1].

(22) $$I=\frac{1}{3}\left(R+G+B\right)$$

(23) $$S=1-\frac{3\times \left[min\left(R,G,B\right)\right]}{\left(R+G+B\right)}$$

(24) $$H=\left\{\begin{array}{c} \theta \;;\;G\geq B\\ 2\pi -\theta ;\;G<B \end{array}\right.$$

where $\theta =\left\{\frac{\left[\left(R-G\right)+\left(R-B\right)\right]/2}{{\left[{\left(R-G\right)}^{2}+\left(R-B\right)\cdot \left(G-B\right)\right]}^{1/2}}\right\}$.

#### 3.3.3. Shape-Based Features

Shape is a crucial low-level property that aids in the recognition of real-world forms and objects. The shape features are retrieved using the methods below.

##### Shape Context

Shape context is a powerful method used in shape-based feature extraction and matching. It is a technique for capturing and describing the shape of an object in a way that is invariant to translation, rotation, and scale.

- Translation

Translation is the process of shifting a shape into a new location in geometry without altering it using any methods.

(25) $$x^{\prime }=x+a$$

(26) $$y^{\prime }=y+b$$

- Scaling

Scaling alters an image’s appearance by resizing it both vertically (y) and horizontally (x). The scaling factors of the x and y dimensions are $S_{x}$ and $S_{y}$.

Scaling for a pixel is given as

(27) $$\left[\begin{array}{c} x\prime \\ y\prime \end{array}\right]=\left[\begin{array}{cc} S_{x} & 0\\ 0 & S_{y} \end{array}\right]\left[\begin{array}{c} x\\ y \end{array}\right]$$

- Rotation

By altering its orientation, rotation changes an image. When rotating counters clockwise by an angle θ, the rotation matrix is

(28) $$\left[\begin{array}{cc} \cos\left(\theta \right) & -sin\left(\theta \right)\\ sin\left(\theta \right) & \cos\left(\theta \right) \end{array}\right]$$

The output obtained when rotating a pixel is given as

(29) $$\left[\begin{array}{c} x\prime \\ y\prime \end{array}\right]=\left[\begin{array}{cc} \cos\left(\theta \right) & -sin\left(\theta \right)\\ sin\left(\theta \right) & \cos\left(\theta \right) \end{array}\right]\left[\begin{array}{c} x\\ y \end{array}\right]$$

The central moments are invariant to translations, and are given as

(30) $$\mu_{pq}=\int \int I\left(x,y\right){\left(x-\bar{x}\right)}^{p}{\left(y-\bar{y}\right)}^{q}dx\;dy$$

Here $\bar{x}=\frac{m_{10}}{m_{00}}\;,\;\bar{y}=\frac{m_{01}}{m_{00}}$

The normalized central moments are invariant to both scaling and translation, and are given as

(31) $$\eta_{pq}=\frac{\mu_{pq}}{\mu_{00}^{\gamma }},\;\gamma =\frac{p+q+2}{2}$$

With $i=0,1,\ldots ,N_{x}-1;j=0,1,\ldots ,N_{y}-1$, the instant for a digital image $I\left(x,y\right)$ is

(32) $$m_{pq}=\sum_{i}\sum_{j}I\left(x,y\right)i^{p}j^{q}$$

##### Fourier Descriptors (FDs)

FDs are a type of shape-based feature extraction method that represents the contour or boundary of a shape using Fourier analysis. The discrete Fourier transform is

(33) $$a_{n}=\frac{1}{N}\sum_{t=0}^{N-1}u\left(t\right)exp\;\left(-\frac{j2\pi nt}{N}\right)$$

$a_{n}$ is the co-efficient, n = 0, 1, …, N − 1, that obtains the FD.

Fourier coefficients of a contour produced are given as

(34) $$a_{n}=exp\left(jn\tau \right)\exp\left(j\varphi \right)\cdot s\cdot {a_{n}}^{o}$$

Here, the $n^{th}$ Fourier coefficient of the original shape is represented by ${a_{n}}^{o}$. The Fourier coefficients that have been normalized are referred to as FDs. By computing the Euclidean distance between their Fractal Dimension representations, the similarity between a query shape Q and a target shape T is calculated. Various u(t) functions are employed to obtain the finite difference equation.

#### 3.3.4. Deep Features Using Pre-Trained Models

Neural networks (NNs) that have been pre-trained on huge datasets provide representations of data, typically images, called deep features retrieved from pre-trained models. High-level semantic information is captured by these representations. Our model makes use of Modified Inception V4 (M-IV4). During training, InceptionV4 incorporates auxiliary classifiers (ACs) at intermediary levels. The second inception block is added before the AC. During training, these classifiers provide regularization and gradient flow. NN intermediate layers are where auxiliary classifiers are included to give gradients during training—an extra path. One way to counteract the vanishing gradient problem is to include ACs. The network can be trained more efficiently as a whole by allowing the gradients from the auxiliary task to circulate back through the network. Regularization stops overfitting and compels the network to learn more beneficial characteristics. Given that the auxiliary goal is distinct from the primary task, the network is motivated to acquire broader and more resilient properties. A residual connection is used to improve Inception-v4 by combining low-level and high-level features, hence increasing the accuracy of the suggested design.

**Figure 3:**
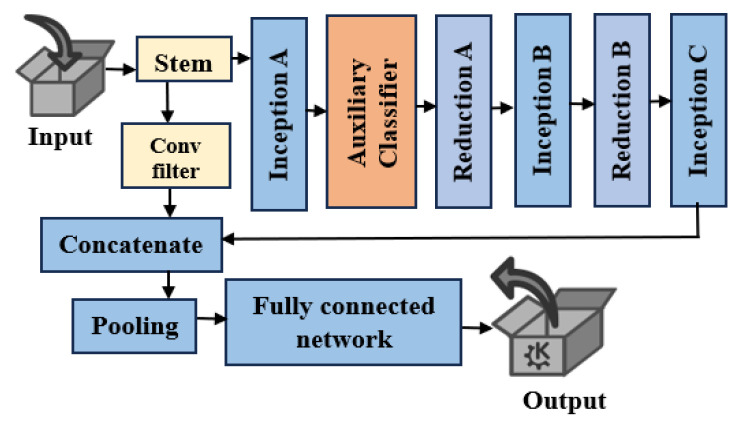
Modified Inception V4.

Usually composed of two or more convolutional layers, a residual block has an identity shortcut (skip connection) that allows it to avoid the convolutional layers. The gradient can pass through the block straight using this skip link, avoiding any non-linear activations, which facilitates the network’s learning of identity mappings. Figure 3 shows the M-IV4 architecture.

(1) Stem: It is responsible for preprocessing an input prior to its entry into the Inception module.
(2) Feature reuse: It constitutes the primary innovation of the suggested paradigm. Utilizing the low-level information, such as color blobs or edges, generated by the initial layers of the CNN, in conjunction with high-level data, might enhance the precision of the framework’s classification. After implementing a 1 × 1 convolution with 512 filters, the suggested approach concatenates the output features from the 3rd convolution in the stem module with the extracted attributes from the I-v4 model.
(3) Inception layers: It enables the internal layers to selectively determine the most appropriate filter size for acquiring the necessary information.
(4) Reduction blocks: The pooling layers are the reduction modules, positioned between the three inception modules.

#### 3.3.5. Feature Selection

The feature selection is achieved by means of the Self-Improved Seagull Optimization Algorithm (SOA) to choose the most pertinent features from a dataset. SOA [24] primarily emulates the migratory and predatory tendencies exhibited by seagulls in their natural habitat. Seagulls’ migration behaviors are characterized by their search for food. The term “attack behaviors” refers to the ways in which seagulls target migratory birds at sea. The algorithm mimics the movements of seagulls as they migrate from one location to another. Among seagulls to avoid collisions, K is utilized to determine the search agent’s position.

(35) $$\vec{V_{s}}=K\times \vec{S_{s}}\left(m\right)$$

$\vec{V_{s}}$ denotes the location of the search agent, $\vec{S_{s}\;}$ is the present location, m is the present iteration, and K is the mobile behavior in the search space.

(36) $$K=f_{c}-\left(m\times \left(\frac{f_{c}}{\max iter}\right)\right);m=0,1,\ldots ,\max iter$$

where $f_{c}$ controls the frequency of the variable.

Upon successfully evading collisions with seagulls, the search agent proceeds into the optimal neighboring direction.

(37) $$\vec{M_{s}}=B\times \left(\vec{S_{bs}}\left(m\right)-\vec{S_{s}}\left(m\right)\right)$$

Here, the location of the search agent $\vec{S_{s}}$ in the direction of the ideal search agent $\vec{S_{bs}}$ is indicated by $\vec{M_{s}}.$ Achieving a balance among exploration and exploitation requires the behavior of B to be stochastic:

(38) $$B=2\times K^{2}\times rd$$

A random number that falls between 0 and 1 is denoted as r_d_.

In relation to the optimal search agent, the search agent can adjust its location.

(39) $$\vec{D_{s}}=\left|\vec{V_{s}}+\vec{M_{s}}\right|$$

The distance between the search agent and the best-fit search agent (i.e., the best seagull with the lower fitness value) is denoted by $\vec{D_{s}}$.

When seagulls attack their prey, they operate in a spiral movement. The following is a statement of the behavior in the x, y, and z planes.

(40) $$x^{\prime }=r\times cos\left(l\right)$$

(41) $$y^{\prime }=r\times sin\left(l\right)$$

(42) $$z^{\prime }=r\times l$$

(43) $$r=g\times e^{lh}$$

The search agent’s updated position is as follows:

(44) $$\vec{S_{s}}\left(m\right)=\left(\vec{D_{s}}\times x^{\prime }\times y^{\prime }\times z^{\prime }\right)+\vec{S_{bs}}\left(m\right)$$

To improve the SOA, we can include an inertia weight $I_{w}$ into the updated location to allow the convergence to be attained faster. Equation (44) can be rewritten as

(45) $$\vec{S_{s}}\left(m\right)=I_{w}\times \left(\vec{D_{s}}\times x^{\prime }\times y^{\prime }\times z^{\prime }\right)+\vec{S_{bs}}\left(m\right)$$

$I_{w}$ can be written as

(46) $$I_{w}=\left(\frac{2}{1+\omega e^{\frac{-2t}{t_{max}}}}-1\right)$$

where t indicates the current iteration, $t_{max}$ is the maximum iteration, ω indicates the controlling factor ranges in (0, 1). Based on these factors, the features are selected and given to the next phase of the model.

### 3.4. Classification

It is the last phase of the procedure. The feature-extracted findings are used to accomplish this output. CNN, optimized-RNN, attention mechanism, and bi-LSTM are all combined in the suggested model to create the Mito-Quartet method for mitosis detection in breast cancer histopathology.

#### 3.4.1. CNN

**Figure 4:**
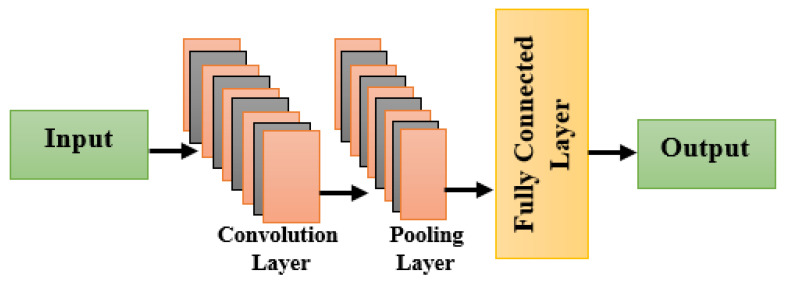
CNN architecture.

It consists of the following layers: input, convolution, pooling, FC, and output. Figure 4 shows the CNN architecture.

##### Input Layer

The input layer is the primary layer of any CNN that accepts images and resizes them before sending them to subsequent layers for processing.

##### CL

To produce a range of feature maps, the input (i/p) cube is convolved with a large number of learnable filters at each CL. The dimension of the input cube is m×n×d. Here, $x_{i}$ for $i^{th}$ feature map of X, d is the count of channels, and m, n is the spatial size. Assume that this layer has k filters, with weights and biases being stated for the $j^{th}$ filter. The CL’s $j^{th}$ o/p is

(47) $$y_{j}=\sum_{i=1}^{d}\hat{f}\left(x\right)\left(m_{i}*w\;j+b\;j\right),j=1,2,\ldots ,k$$

f → activation function.

It is given as

(48) $$\sigma \left(m\right)=\max\left(0,m\right)$$

##### Pooling Layer

Due to the existence of repetitive information in images, these are sometimes introduced after numerous CL. The pooling process causes the spatial size of the feature maps to steadily decrease, and the computation and parameter count of the network also decrease. With a p × p window-size neighbor denoted as S, the standard pooling technique can be shown as follows:

(49) $$Z=\frac{1}{F}\sum_{i,j\epsilon S}m_{i,j}$$

The count of elements in S is denoted as F.

The activation value for location (i, j) is denoted as $m_{i,j}.$

##### FC Layers

In order to extract in-depth and abstract information, the features from the previous stage are sent to this step.

##### Output Layer

The network’s output is driven by this layer.

#### 3.4.2. Optimized-RNN

**Figure 5:**
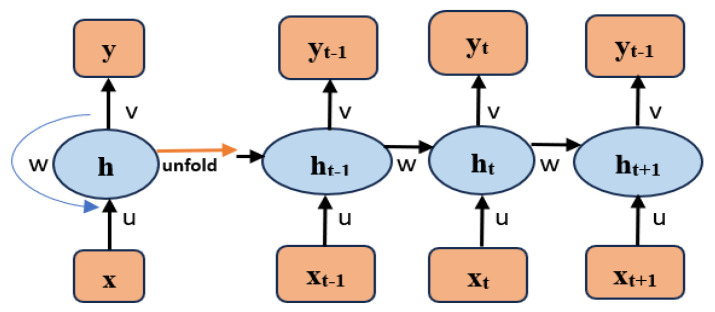
RNN structure.

It is a neural network in which the output from the previous stage is used as the input for the current model. Normally, inputs and outputs of the Neural Network NN are unrelated to one another. However, when predicting a sentence’s next word, preceding words need to be retained. The created RNN uses a hidden layer to address this issue. The hidden state is the primary and most significant aspect of an RNN, which contains certain sequence-related information. Figure 5 shows the RNN structure.

The current state is

(50) $$h_{t}=f\left(h_{t-1},\;x_{t}\right)$$

Here, $h_{t-1}$ is the prior state and $h_{t}$ is the current state. $x_{t}\;is\;the\;input\;state$.

Applying activation in the above equation, it can be written as

(51) $$h_{t}=\tan h\left({wh}_{t-1},+ux_{t}\right)$$

where tanh is the activation function.

The network o/p is given as

(52) $$y_{t}=v\cdot h_{t}$$

where v is the weights for the link of the hidden-layer-to-output-layer layer.

The vanishing gradient problem is a common issue with RNNs, which can make it difficult for them to identify long-range connections in sequential data. To solve this problem, GRUs (Gated Recurrent Units) have been created; these are regarded as improved RNN variations. The capacity and capability of the network to capture complicated data are increased. Gates are used by GRUs to regulate information flow. Additionally, a GRU performs better on smaller datasets. The $u_{t}$ and $q_{t}$ are the only gates present in the GRU structure. The $q_{t}$ manages the power of $v_{t-1}$ on the output of the current state, whereas the reset gate manages its effect on the input of the current moment. As follows is the GRU formula:

(53) $$q_{t}=\sigma \left(W_{q}\cdot \left[v_{t-1},x_{t}\right]+b_{q}\right)$$

(54) $$u_{t}=\sigma \left(W_{u}\cdot \left[v_{t-1},x_{t}\right]+b_{u}\right)$$

(55) $$vˆ\;t=f\left(W_{v}\cdot \left[u_{t}\cdot v_{t-1},x_{t}\right]+b_{v}\right)$$

(56) $$v_{t}=\left(\left(1-q\right)\cdot v_{t-1}+q_{t}\cdot vˆ\;t\right)$$

$u_{t}$ → reset gate, $q_{t}$ → update gate, $x_{t}$ → input information, σ → sigmoid function, $v_{t-1}$ → previous hidden state, and f → Tanh activation function.

- From (52), how much of the $v_{t-1}$ must be maintained is decided by the $q_{t}$. It uses a sigmoid function to transfer the current input, along with the $v_{t-1}$. The sigmoid function produces an output that lies between 0 and 1. In this instance, 0 denotes complete forgetting of the prior state, and 1 denotes complete retention of the prior state.
- From (53), the $u_{t}$ determines the count of the previous state that needs to be reset or forgotten. Similar to the update gate, this also accepts the current input and $v_{t-1}$, passes them using a sigmoid function, and outputs the reset gate.
- From (54), the new hidden state candidate that is determined using $u_{t}$ and the current input is known as the candidate hidden state. $v_{t-1}$ is controlled by the rest gate and combined with the present input state. The output of the tanh activation function is the result, which yields a value in the range between −1 and 1.
- From (55), finally, based on the update gate, the previous and candidate hidden states are used to compute the hidden state. The model must keep the majority of the previously hidden state if the output of the $q_{t}$ is nearer to 1, and it prefers to use the majority of the candidate hidden state if it is nearer to 0. This causes the GRU to update its hidden state.

The self-attention mechanism receives the output of the GRU layer after the data has been processed by the dual GRU layers. It computes the weighted representations of the GRU outputs, by which it selects the most relevant data.

#### 3.4.3. Attention Mechanism

The ability of a framework to assess the relative weights of various features in its input sequence while generating estimates is known as self-attention.

The self-attention mechanisms (SAMs) have an attention (A) function that operates simultaneously or sequentially to capture various components of the input data. Each SAM calculates attention scores and produces outputs that are weighted based on the data that is received. The two halves of the i/p sequence are the query sequence (Q) and the key sequence (K). Q is multiplied by the weight matrix to obtain a set of attention weights. To obtain the output values (V), the K is weighted using the attention weights, and the weighted K is then multiplied by the weight matrix a second time.

(57) $$A\;score=y\cdot W_{Q1}\cdot {\left(y\cdot W_{K1}\right)}^{T}$$

(58) $$A\;weight=Softmax\left(A1\;score\right)$$

(59) $$A\;output=A1\;weight\cdot \left(y\cdot W_{V1}\right)$$

$W_{Q1},W_{K1},W_{V1}$ → learnable weight matrices for Attention.

#### 3.4.4. Bi-LSTM

The majority of the LSTM Neural Network (NN) is composed of three gated units: forget gate $f_{t}$, input gate $i_{t}$, and output gate $o_{t}$. Sequences can be processed in both ways by Bi-LSTM. Two LSTM networks process the sequence, one from right to left and the other from left to right. To enhance the model’s effectiveness, a Bi-LSTM usually concatenates the hidden states from both left-to-right and right-to-left LSTM networks. This allows for capturing both the past and future of each element in the context of the sequence. Specialized gated units are used to gather and store the sequence data to preserve the interdependence of time series data over long distances and accomplish precise prediction. Then, $i_{t}$ processes a considerable portion of the supplied data. $f_{t}$ is necessary for the current neuron to retain past information. The output gate provides the neuron’s o/p.

Assuming that the i/p sequence is $x_{1},x_{2},\ldots ,x_{t}$, each LSTM neuron parameter at time t can be computed as

(60) $$i_{t}=S\left(W_{i}*\left[h_{t-1},x_{t}\right]\right)$$

(61) $$f_{t}=S\left(W_{f}*\left[h_{t-1},x_{t}\right]\right)$$

(62) $$o_{t}=S\left(W_{o}*\left[c_{t},h_{t-1},x_{t}\right]\right)$$

(63) $$c_{t}=f_{t}*C_{t-1}+i_{t}*tanh\left(W_{c}*\left[h_{t-1},x_{t}\right]\right)$$

(64) $$h_{t}=o_{t}*tanh\left(c_{t}\right)$$

A predetermined process is used to optimize the weights between the nodes in the LSTM NN, which can enhance the framework’s ability to generalize and predict by rationalizing the weights between neurons.

#### 3.4.5. Integration Layer

This layer integrates the outputs from the CNN, RNN, AM, and Bi-LSTM.

#### 3.4.6. FC Layers

Following integration, integrated attributes are often passed through one or more fully connected (FC) layers.

#### 3.4.7. Output Layer

Class probabilities are generated by the final output layer using an activation function, commonly SoftMax. The total number of classes in the classification issue corresponds to the total number of neurons in this layer. With a vector of raw scores or logits as input, it creates a probability distribution over the classes. The chance that a given data point belongs to each class is calculated by the SoftMax function. The final class label is anticipated to be the one with the highest likelihood.

(65) $$Softmax\left(y\right)=\frac{e^{y}}{\sum_{j}e^{yj}}$$

## 4. Experimental Results and Analysis

Performance metrics are utilized to analyze the efficiency of the proposed approach on the MITOSIS 2012 benchmark dataset. To ensure fairness and comparability, all the baseline models, whether it is RNN, Bi-LSTM, GRU, CNN, or MLP, were implemented and conducted under the same testing conditions on the same system platform. The achieved results verify the superiority of the proposed system within various performance metrics.

### 4.1. Dataset Description

Performance evaluation of the suggested mitosis detection method was carried out on the dataset of the ICPR 2012 MITOSIS Contest. This dataset contains five whole slide images of biopsies in the breast stained with hematoxylin and eosin (H&E) dyes, which were annotated independently by two expert pathologists. Ten high-power fields (HPFs) were chosen at a magnification of 40× on each biopsy image, providing a total of 50 annotated HPFs.

The high spatial resolution associated with the whole slide images required the use of the patch-based approach, in which the HPFs are further partitioned into smaller image patches that include the mitotic and non-mitotic areas. To avoid any form of bias, the patches within each HPF would only be associated with one subset in the training and evaluation process. The complete dataset is openly available in the MITOSIS 2012 repository [27].

### 4.2. Implementation Details

All experiments were implemented using MATLAB R2023a. The models were trained using the Adam optimizer with an initial learning rate of 0.0001, batch size of 16, and 100 epochs. Early stopping was applied to prevent overfitting. The experiments were conducted on a system equipped with an Intel Core i7 CPU, 32 GB RAM, and an NVIDIA RTX 3080 GPU (10 GB).

### 4.3. Performance Evaluation

Performance metrics are used to evaluate current and suggested methods. The values of the evaluation metrics for the suggested and current approaches are shown in Table 1.

#### 4.3.1. Accuracy

Accuracy measures the proportion of correct predictions among all input observations. The calculation is performed using the following formula:

(66) $$Accuracy=\frac{Number\;of\;correct\;predictions}{Total\;number\;of\;samples}$$

**Figure 6:**
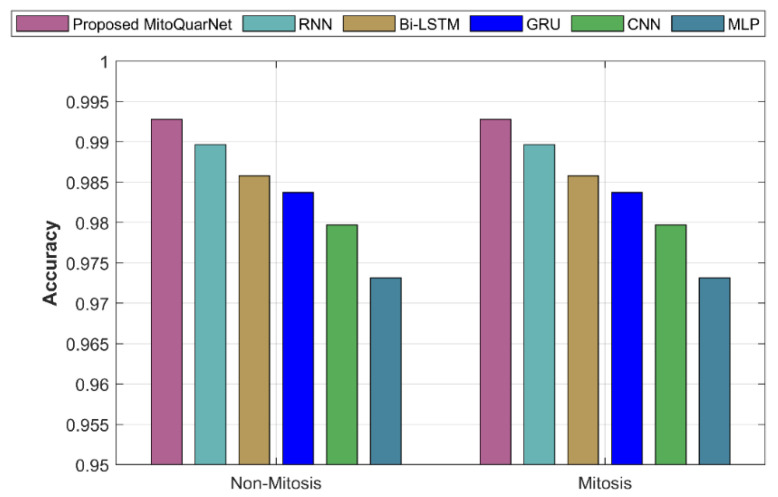
An examination of the proposed and existing approaches with respect to accuracy.

Figure 6 shows the analysis of the suggested and implemented approaches in terms of accuracy. According to the graph, the suggested strategy has an accuracy of 99.2%, whereas the current approaches have a lower accuracy.

#### 4.3.2. Sensitivity

Sensitivity measures the percentage of true positives (TPs) that are precisely detected.

(67) $$Sensitivity=\frac{TP}{TP+FN}$$

**Figure 7:**
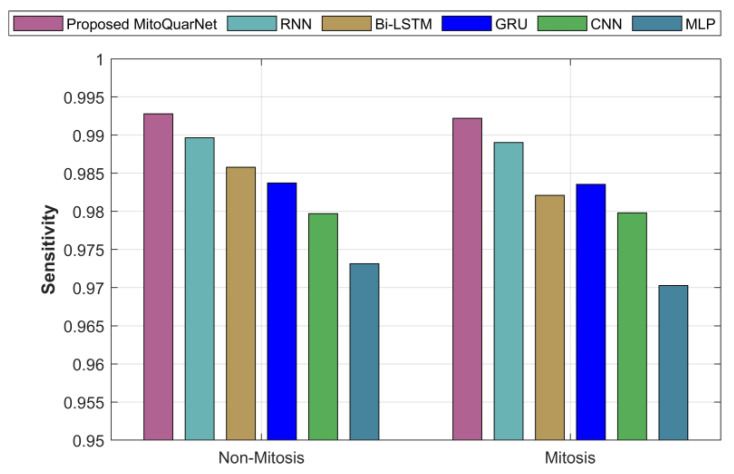
An examination of the proposed and existing approaches with respect to sensitivity.

The recommended and used procedures in relation to sensitivity are examined in Figure 7. According to the graph, the present methods are less sensitive than the suggested approach, which has a sensitivity of nearly 99.2%.

#### 4.3.3. Specificity

Specificity measures the proportion of true negatives. It is calculated using

(68) $$Specificity=\frac{TN}{TN+FP}$$

**Figure 8:**
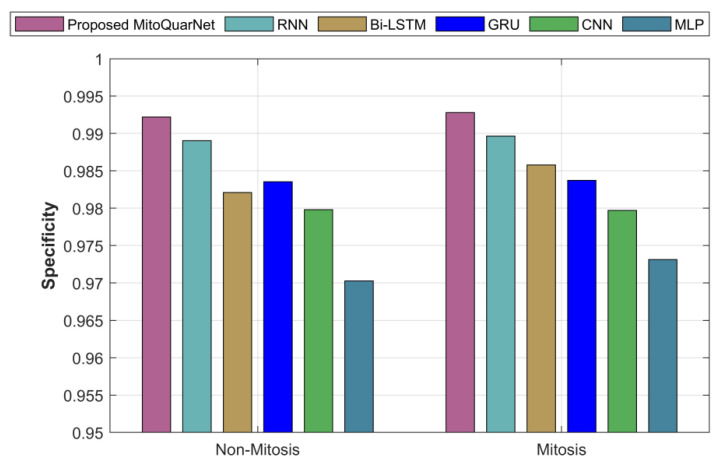
Analysis of recommended and current methods by specificity.

The analysis of the suggested and actual procedures in terms of specificity is presented in Figure 8. The present methods have a lower specificity than the suggested strategy, which has a specificity of around 99.3%, according to the graph.

#### 4.3.4. F Measure

A combination statistic that combines two evaluation metrics to provide a general score for performance evaluation is as follows:

(69) $$F\text{\_}measure=2*\frac{Precision*Recall}{Precision+Recall}$$

**Figure 9:**
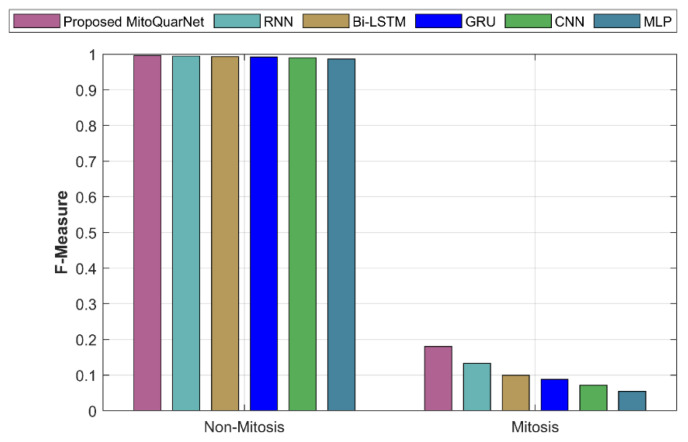
Analysis of recommended and current methods by F-measure.

The analysis of the suggested and current approaches with respect to F-measure is provided in Figure 9. According to the graph, the current methods have a lower F-measure than the suggested approach, which has an F-measure of around 99.6%, according to the graph.

#### 4.3.5. False Negative Rate (FNR)

It describes the values that are actually positive but predicted to be negative.

(70) $$FNR=\frac{FN}{FN+TP}$$

**Figure 10:**
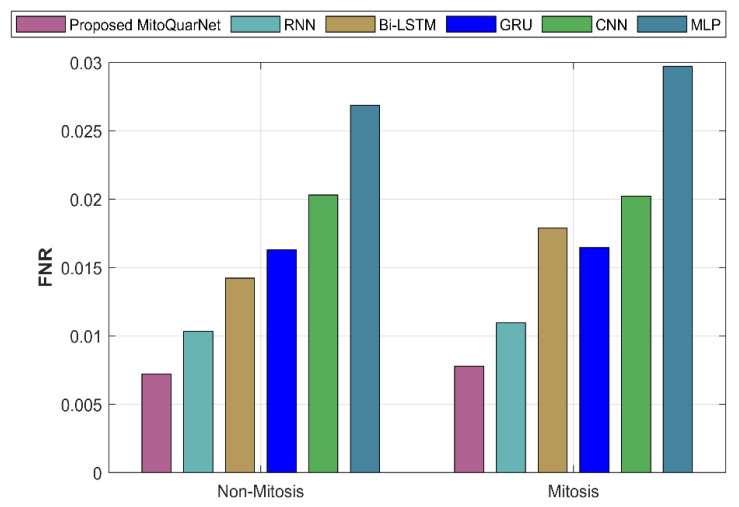
Analysis of recommended and current methods by FNR.

The analysis of the suggested and current approaches with respect to FNR is presented in Figure 10. According to the graph, the FNR of the current methods is higher than that of the suggested strategy, which is 0.007224.

#### 4.3.6. False Positive Rate (FPR)

It describes the values that are expected to be positive but are actually negative.

(71) $$FPR=\frac{FP}{FP+TN}$$

**Figure 11:**
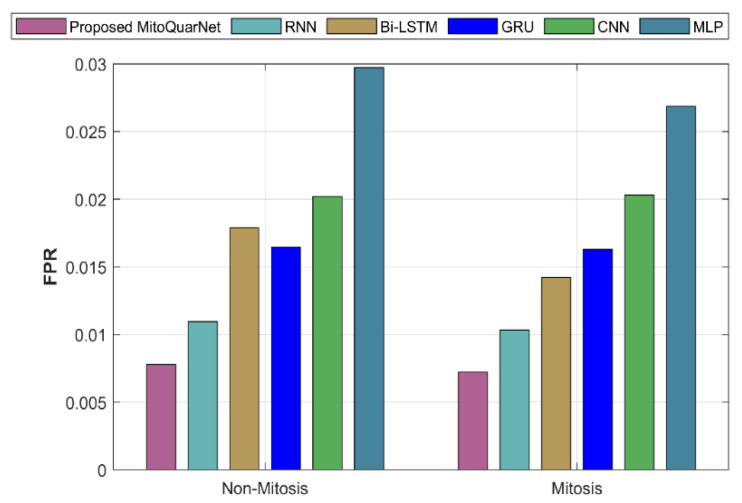
Analysis of recommended and current methods by FPR.

The analysis of the suggested and current methods by FPR is presented in Figure 11. According to the graph, the suggested method has an FPR of 0.006347, whereas the current methods have a higher FPR.

#### 4.3.7. Fowlkes–Mallows’ Index (FMI)

The Fowlkes–Mallows index is an external assessment technique utilized to quantify the resemblance in segmenting.

(72) $$FPR=\sqrt{\frac{TP}{TP+FP}\cdot \frac{TP}{TP+FN}}$$

**Figure 12:**
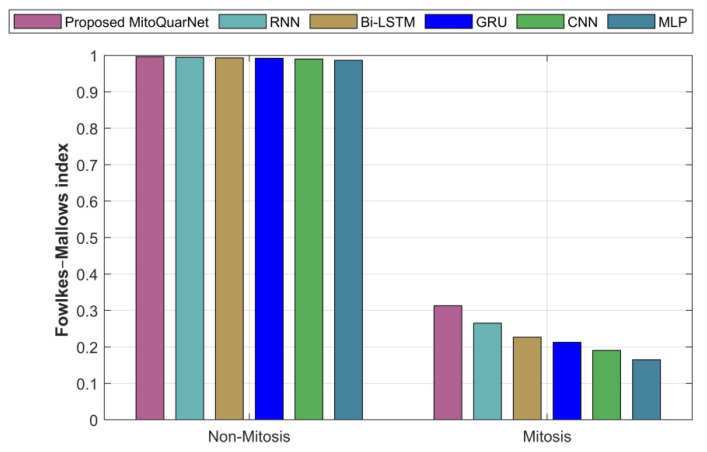
Analysis of recommended and current methods by FMI.

Figure 12 shows the examination of the suggested and current methods by FMI. According to the graph, the suggested method has an FMI of 0.996379, whereas the current methods have lower values.

#### 4.3.8. Markedness

It is a quantitative assessment of the reliability of both positive and negative predictions made by a system.

**Figure 13:**
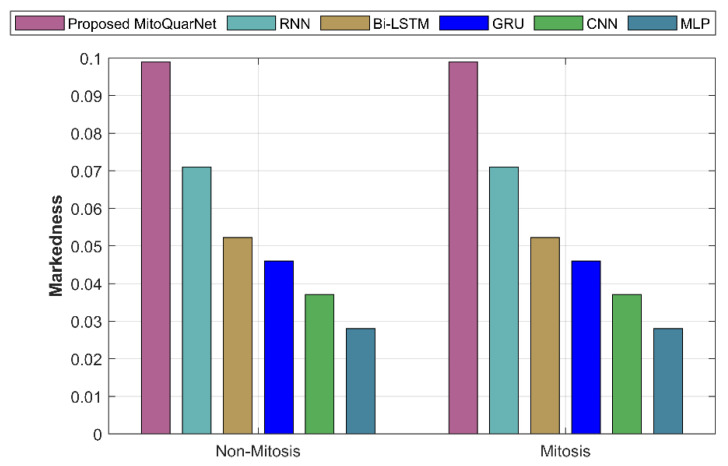
Analysis of recommended and current methods by markedness.

The recommended and current strategies by markedness are examined in Figure 13. According to the graph, the suggested strategy has a markedness of 0.09898, while the current approaches have lower values.

The suggested approach achieves the best accuracy of 99.28% among the considered approaches, demonstrating the efficiency of integrating deep learning with handcrafted features and the optimal selection of features. Additionally, the state-of-the-art approaches based on deep learning, such as the hybrid CNN [16], the graph regularized DenseNet [19], and the CNN feature fusion [21], result in a slight accuracy decrease, while the accuracy of the traditional SVC [22] approach is the lowest among all. Table 2 presents the performance of the proposed model using five-fold cross-validation with detection-relevant metrics to evaluate robustness and generalization across different data splits.

The results in Table 3 show consistent and stable performance across all folds, with high sensitivity, specificity, precision, and F1-score, indicating reliable mitosis detection. The low average false positive rate (0.0063) and false negative rate (0.0072) further confirm effective error control and reduced overfitting, demonstrating the strong generalization capability of the proposed framework.

### 4.4. Ablation Study

An ablation study was conducted to evaluate the individual contribution of each component in the proposed mitosis detection framework. By systematically removing or replacing key modules while keeping the remaining architecture unchanged, the impact of preprocessing, ROI localization, feature extraction, optimization, and classification stages on detection performance was analyzed.

Table 4 presents the ablation study results using detection-relevant metrics. The configuration without preprocessing shows a noticeable reduction in precision, recall, and accuracy, indicating that normalization and enhancement steps play a crucial role in improving feature quality. Removing the modified FCN for ROI identification further degrades performance, confirming the importance of accurate region localization in reducing false detections. The absence of deep feature extraction leads to the lowest recall and highest false negative rate, highlighting its significance in capturing discriminative mitotic patterns. When the SA-SOA optimization module is excluded, a clear drop in F1-score and increased false positives are observed, demonstrating its effectiveness in selecting optimal features. Replacing the Mito-Quartet classifier with a simple CNN results in moderate performance loss, suggesting that the hybrid classifier better captures complex spatial and contextual relationships. Overall, the proposed full model achieves the highest precision (0.9964), recall (0.9927), F1-score (0.9964), and accuracy (0.9928), with the lowest FPR and FNR, confirming that each component contributes positively to the final detection performance.

### 4.5. Discussion

The proposed Quadra Sense framework demonstrates strong and consistent performance on the MITOSIS 2012 dataset, as evidenced by the five-fold cross-validation results, which indicate stable generalization and minimal overfitting across different data splits. The inclusion of detection-relevant metrics further highlights the model’s ability to accurately identify mitotic figures while maintaining low false positive and false negative rates. The conducted ablation study clearly shows that each component of the framework plays an important role in overall performance. Specifically, preprocessing enhances feature quality, the modified FCN effectively localizes regions of interest and reduces background noise, and deep feature extraction captures discriminative mitotic patterns. The SA-SOA optimization module improves feature selection, while the Mito-Quartet classifier strengthens decision-making by modeling complex feature relationships.

From an implementation perspective, the modular and hybrid design of the proposed system balances accuracy and computational efficiency. The integration of deep learning with handcrafted texture, color, and morphological features enhances robustness, particularly under limited data conditions. Although the experimental evaluation was limited to a single benchmark dataset, this limitation is explicitly acknowledged, as differences in staining, scanner characteristics, and annotation strategies may affect generalization. Compared with purely deep learning–based approaches that rely on large-scale datasets and high computational resources, the proposed hybrid framework achieves competitive performance with improved adaptability. Future work will focus on external dataset validation and cross-dataset analysis to further assess robustness and clinical applicability.

## 5. Conclusions

Early detection is essential because breast cancer is thought to be the second most prevalent cause of cancer-related death among women. Early detection is difficult because of the existing detection tools’ shortcomings in objectivity and accuracy. Quadra Sense, a combination of deep learning (DL) classifiers for mitosis detection in breast cancer histopathology, is proposed to address the shortcomings of current approaches. It has a higher potential to produce higher accuracy levels. With an accuracy of 99.2%, sensitivity of 99.2%, F-measure of approximately 99.6%, specificity of approximately 99.3%, FPR of 0.006347, FNR of 0.007224, FMI of 99.6%, and markedness of 98.8%, the suggested strategy is highly recommended. The results demonstrate that, when compared to the current techniques, the suggested approach is superior. Despite the promising result obtained, the experimental evaluation conducted within this study only focused on the benchmark dataset provided by the MITOSIS 2012 challenge. This is because differences in annotation schemes, staining differences, and evaluation metrics make it difficult to conduct direct validation on larger benchmarks such as the MITOSIS 2014 and TUPAC16 challenges. In future research, evaluation on the respective challenges will be conducted to further investigate generalization performance and real-world applicability.

## Figures and Tables

**Table 1:** Evaluation metric values for the proposed and existing methods.

Technique	Sen	Spec	Acc	F-Measure	FPR	FNR	FMI	Markedness
Proposed	0.9927	0.9937	0.9928	0.9964	0.0063	0.0072	0.9964	0.0990
RNN	0.9896	0.9890	0.9897	0.9948	0.0110	0.0103	0.9948	0.0710
Bi- LSTM	0.9857	0.9856	0.9858	0.9928	0.0144	0.0142	0.9929	0.0524
GRU	0.9837	0.9827	0.9837	0.9918	0.0173	0.0163	0.9918	0.0459
CNN	0.9796	0.9778	0.9797	0.9897	0.0222	0.0203	0.9898	0.0370
MLP	0.9731	0.9720	0.9731	0.9864	0.0280	0.0269	0.9865	0.0280

**Table 2:** Comparative analysis of proposed method with existing techniques.

Technique/Reference	Methodology	Accuracy
Proposed	Hybrid DL + handcrafted features, Modified FCN, M-IV4, SA-SOA, Mito-Quartet	0.9928
Sampath & Srinath [16]	Hybrid CNN with SCA-based optimization	0.969
Patel et al. [19]	DenseNet + graph-based adaptive regularization	0.991
Al-Jabbar et al. [21]	CNN + handcrafted feature fusion	0.987
Ramkumar [22]	SVC-based ML framework	0.941

**Table 3:** Five-fold cross-validation performance with detection-relevant metrics.

Fold	Sensitivity	Specificity	Precision	Accuracy	F1-Score	FPR	FNR
Fold-1	0.9918	0.9929	0.9959	0.9919	0.9958	0.0071	0.0082
Fold-2	0.9924	0.9935	0.9961	0.9923	0.9961	0.0065	0.0076
Fold-3	0.9931	0.9942	0.9966	0.9930	0.9966	0.0058	0.0069
Fold-4	0.9920	0.9931	0.9960	0.9921	0.9960	0.0069	0.0080
Fold-5	0.9939	0.9947	0.9970	0.9938	0.9970	0.0053	0.0061
Average	0.9927	0.9937	0.9964	0.9928	0.9964	0.0063	0.0072

**Table 4:** Ablation study results with detection-relevant metrics.

Configuration	Precision	Recall	F1-Score	Specificity	Accuracy	FPR	FNR
Without Preprocessing	0.9705	0.9612	0.9721	0.9684	0.9645	0.0316	0.0388
Without Modified FCN (ROI)	0.9759	0.9689	0.9764	0.9727	0.9703	0.0273	0.0311
Without Deep Feature Extraction	0.9681	0.9578	0.9690	0.9649	0.9613	0.0351	0.0422
Without SA-SOA Optimization	0.9798	0.9746	0.9817	0.9783	0.9764	0.0217	0.0254
Without Mito-Quartet (Simple CNN)	0.9854	0.9815	0.9873	0.9846	0.9829	0.0154	0.0185
Proposed (Full Model)	0.9964	0.9927	0.9964	0.9937	0.9928	0.0063	0.0072

## Data Availability

The original data presented in the study are openly available in the 2012 MITOSIS Contest Dataset, hosted on GitHub, at https://mitos-atypia-14.grand-challenge.org/ (accessed on 23 November 2025).
